# Inositol in the MAnaGemENt of abdominal aortic aneurysm (IMAGEN): study protocol for a randomised controlled trial

**DOI:** 10.1186/s13063-017-2304-x

**Published:** 2017-11-16

**Authors:** Sophie E. Rowbotham, Jenna L. Pinchbeck, Georgina Anderson, Bernie Bourke, Michael Bourke, T. Christian Gasser, Rene Jaeggi, Jason S. Jenkins, Corey S. Moran, Susan K. Morton, Christopher M. Reid, Ramesh Velu, Lisan Yip, Joseph V. Moxon, Jonathan Golledge

**Affiliations:** 1 School of Medicine, The University of Queensland, Herston, QLD 4006 Australia; 2 Department of Vascular Surgery, The Royal Brisbane and Women’s Hospital, Herston, QLD 4029 Australia; 3 Queensland Research Centre for Peripheral Vascular Disease, College of Medicine and Dentistry, James Cook University, Townsville, QLD 4811 Australia; 4 Gosford Vascular Services, Gosford, NSW 2250 Australia; 5 Department of Solid Mechanics, School of Engineering Sciences, KTH Royal Institute of Technology, 100 44 Stockholm, Sweden; 6 School of Public Health, Curtin University, Perth, WA 6000 Australia; 7 School of Public Health and Preventive Medicine, Monash University, Melbourne, VIC 3004 Australia; 8 Department of Vascular and Endovascular Surgery, The Townsville Hospital, Townsville, QLD 4811 Australia

**Keywords:** Abdominal aortic aneurysm, Inositol, Clinical trial

## Abstract

**Background:**

An abdominal aortic aneurysm (AAA) is a focal dilation of the abdominal aorta and is associated with a risk of fatal rupture. Experimental studies suggest that myo-inositol may exert beneficial effects on AAAs through favourable changes to biological pathways implicated in AAA pathology. The aim of the Inositol in the MAnaGemENt of abdominal aortic aneurysm (IMAGEN) trial is to assess if myo-inositol will reduce AAA growth.

**Methods/design:**

IMAGEN is a multi-centre, prospective, parallel-group, randomised, double-blind, placebo-controlled trial. A total of 164 participants with an AAA measuring ≥ 30 mm will be randomised to either 2 g of myo-inositol or identical placebo twice daily for 12 months. The primary outcome measure will be AAA growth estimated by increase in total infrarenal aortic volume measured on computed tomographic scans. Secondary outcome measures will include AAA diameter assessed by computed tomography and ultrasound, AAA peak wall stress and peak wall rupture index, serum lipids, circulating AAA biomarkers, circulating RNAs and health-related quality of life. All analysis will be based on the intention-to-treat principle at the time of randomisation. All patients who meet the eligibility criteria, provide written informed consent and are enrolled in the study will be included in the primary analysis, regardless of adherence to dietary allocation.

**Discussion:**

Currently, there is no known medical therapy to limit AAA progression. The IMAGEN trial will be the first randomised trial, to our knowledge, to assess the value of myo-inositol in limiting AAA growth.

**Trial registration:**

Australian New Zealand Clinical Trials Registry, ACTRN12615001209583. Registered on 6 November 2015.

**Electronic supplementary material:**

The online version of this article (doi:10.1186/s13063-017-2304-x) contains supplementary material, which is available to authorized users.

## Background

Abdominal aortic aneurysm (AAA) is a focal dilation of the abdominal aorta with a maximum diameter ≥ 30 mm [1]. The most recognised complication of AAA is aortic rupture, the risk of which increases at larger diameters [1, 2]. Patients with AAA have at least a twofold increased rate of cardiovascular events, particularly myocardial infarction, compared with individuals with normal aortic diameter [3]. AAAs can be readily identified when they are small by ultrasound surveillance or incidental imaging. At this stage, patients have minimal risk of AAA rupture and can thus potentially be treated early [4–7]. There are, however, limited current means to effectively treat such patients [1, 8, 9]. The only treatment currently available for AAA is surgical repair, which is associated with significant mortality (1–5%), peri-operative complications, in-hospital costs, and the need for post-operative follow-up, imaging and, in some cases, repeat surgery [1, 5, 7, 10–12]. Four randomised controlled trials have demonstrated that early elective surgery does not reduce mortality for patients with small (<55 mm) AAAs [4–7]. It is estimated that 50–70% of small AAAs will eventually grow to a size at which surgical repair is indicated [5, 7, 13]. Effective medical therapies administered to patients with small AAAs could prevent or limit AAA growth, thereby reducing or avoiding the need for surgery. Only a small number of randomised controlled trials have been carried out to assess medical therapies for AAA, and none have identified an effective treatment [8, 9, 14]; however, a number of such trials are ongoing [15].

Findings from previous studies suggest that increased consumption of myo-inositol has the potential to target key pathways implicated in AAA progression [16–19]. Myo-inositol can be ingested from a number of foods and is found in high concentrations in vegetables, nuts, whole grains and fresh fruits [20]. Consumption of these foods has been associated with reduced prevalence of AAA and reduced incidence of cardiovascular events [21, 22]. Myo-inositol is a key component of the cell membrane structure and cell signalling pathways [23–25]. Myo-inositol and its metabolites play an important role in the control of cell proliferation, cell survival, matrix remodelling and inflammation [23–31]. Inositol phosphates have been shown to inhibit nuclear factor kappa-light-chain-enhancer of activated B cells (NF-κB) activation, which is recognised as a master regulator of inflammation and matrix remodelling [26]. Activation of NF-κB upregulates chemokines such as C-C motif chemokine 22 (CCL22), matrix metalloproteinases (MMPs) and cathepsins, which are all implicated in AAA pathogenesis [32–37]. Inhibition of NF-κB has previously been demonstrated to inhibit AAA growth in pre-clinical animal models [33, 36–38]. Inositol phosphates also control the mechanistic target of rapamycin (mTOR) signalling pathway [39]. Inhibition of mTOR limits AAA growth in pre-clinical rodent models by reducing aortic recruitment of inflammatory cells and inhibiting production of pro-inflammatory cytokines [40, 41]. Inositol phosphates have also been shown to favourably influence the production of bone proteins [42]. It has previously been reported that bone proteins, including osteoprotegerin (OPG), osteopontin (OPN) and sclerostin (SOST), are implicated in the pathogenesis of AAA [34, 35, 43, 44]. Collectively, these data suggest that myo-inositol has potential ability to limit key pathological mechanisms involved in AAA, although the effect of myo-inositol administration on AAA growth has not previously been assessed.

Dietary supplementation with myo-inositol has previously been examined in a number of patient groups, including women with polycystic ovary syndrome and gestational diabetes, as well as patients with depression, with favourable results [16, 17, 19, 45, 46]. Administration of doses of ~ 4 g per day has been reported to reduce circulating concentrations of pro-inflammatory cytokines such as resistin, as well as to favourably modify circulating lipids by reducing triglycerides and raising high-density lipoprotein (HDL) cholesterol and phosphatidylcholine plasmalogen lipids [16–19]. It has previously been reported that patients with AAA have a distinct circulating pro-inflammatory and lipid profile [47–50]. AAA is associated with high serum resistin, triglycerides, linoleic acid-containing triacylglycerols and diacylglycerols, and low serum HDL cholesterol [47–50]. Given the ability of myo-inositol to reduce inflammation and favourably modify the lipid profile in other patients, it is possible that this approach could have similar effects in patients with AAA. Such effects would be expected to limit the main complications in these patients, specifically AAA growth and cardiovascular events.

The primary aim of the Inositol in the MAnaGemENt of abdominal aortic aneurysm (IMAGEN) trial is to determine if dietary supplementation with 2 g of myo-inositol twice daily limits AAA growth over 12 months, evidenced by limiting AAA volume assessed on the basis of computed tomography (CT). The secondary aims of the IMAGEN trial are to examine the effect of dietary supplementation with 4 g of myo-inositol daily for 12 months on (a) AAA growth assessed by aortic diameter measured by CT and ultrasound; (b) AAA peak wall stress (PWS) and peak wall rupture index (PWRI) estimated on the basis of CT; (c) lipid profile; (d) circulating markers of inflammation, matrix remodelling and circulating RNA profile; and (e) health-related quality of life.

## Methods/design

### Study design

IMAGEN is a multi-centre, prospective, parallel-group, randomised, double-blind, placebo-controlled trial designed to assess if dietary supplementation with 2 g of myo-inositol twice daily for 12 months will reduce AAA growth. The trial will be conducted at four sites in Australia: The Royal Brisbane and Women’s Hospital, Brisbane; Gosford Vascular Services, Gosford; The Townsville Hospital, Townsville; and Mackay Base Hospital, Mackay. This trial protocol is reported according to the Standard Protocol Items: Recommendations for Interventional Trials (SPIRIT) (*see* Additional file 1). Only research personnel who are directly involved in the recruitment and data collection aspect of the study will have access to participants’ personal details. All case report forms (CRFs), source documentation and samples will be de-identified and coded with a study number, according to good clinical practice (GCP) guidelines.

### Participants

**Fig. 1:**
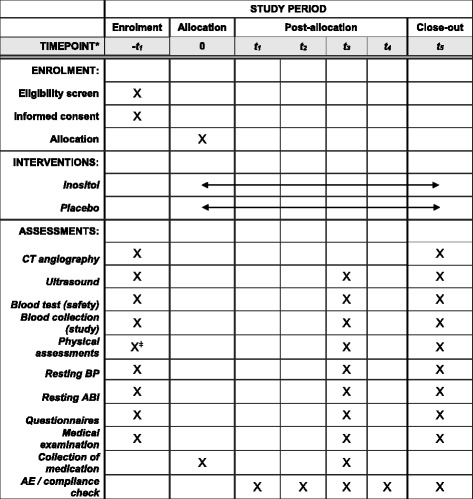
Standard Protocol Items: Recommendations for Interventional Trials (SPIRIT) figure: schedule of enrolment, interventions, and assessments. *ABI* Ankle-brachial index, *BP* Blood pressure, *CT* Computed tomography

The IMAGEN trialists plan to enrol 164 participants who have an asymptomatic infrarenal AAA measuring a maximum diameter ≥ 30 mm on the basis of CT or ultrasound, both of which will be performed as part of the baseline assessments (see Fig. 1). Participants will be included only if they have a high likelihood of treatment compliance over 12 months as determined by their treating physician or local study coordinator. IMAGEN will not include patients with an existing indication for AAA repair or expectation that this will be revised within 12 months. Additional exclusion criteria include previous reaction to myo-inositol supplementation, inability to tolerate abdominal imaging, inability to provide blood samples and previous abdominal aortic surgery. A full list of inclusion and exclusion criteria is given in Table 1.

**Table 1:** Patient eligibility criteria

Inclusion criteria
• Ability to provide written informed consent
• Diagnosis with an infrarenal AAA measuring a maximum diameter ≥ 30 mm based on CT or ultrasound
• No current indication for AAA repair according to the treating physician or expectation that this will be revised over 12 months
• High likelihood of treatment compliance over 12 months according to the treating physician and local study coordinator
• Aged ≥ 50 years
Exclusion criteria
• Symptomatic, ruptured or mycotic AAA
• Contraindication to the study protocol:
○ Previous reaction to myo-inositol supplementation
○ Inability to tolerate abdominal imaging
○ Inability to provide blood samples for safety and outcome analysis
• Previous abdominal aortic surgery
• Current or planned participation in another randomised trial
• Treating physician feels the patient is not suitable for trial entry

*CT* Computed tomography, *AAA* Abdominal aortic aneurysm

### Randomisation

The randomisation scheme will be generated from Stata code (StataCorp, College Station, TX, USA) and maintained by the central clinical trial pharmacist. Randomisation to myo-inositol or placebo will be stratified by study centre, presence of diabetes and aortic diameter (30–34, 35–39, 40–44, 45–49 and ≥ 50 mm) as measured on ultrasounds. Randomisation will be blocked in a 1:1 ratio (randomly to a maximum block size of 8). The central and site trial managers will be blinded to treatment allocation. Eligible and consenting participants will be allocated to inositol or placebo groups via a telephone call. Once an allocation number is obtained, the trial supplement will be dispensed by the local clinical trial pharmacist (after randomisation and at the 6-month time point). Allocation concealment will be achieved by using a central randomisation centre and employing identical packaging of intervention and placebo. Participants, investigators and outcome assessors will be blinded to treatment allocation. In the case of an emergency where breaking of the group allocation blinding is required, the local study centre clinical trial pharmacist will be contacted.

### Interventions

Participants in the intervention group will receive four 500-mg capsules of myo-inositol, which is to be taken orally at 08:00 and 20:00 daily. This dose has been used in previous clinical trials in other patient groups and is well tolerated over 12 months, with no side effects reported [16–19, 45]. Furthermore, this dose has been reported to favourably modify lipid profile and reduce circulating concentrations of pro-inflammatory cytokines in other patient groups [16–19, 45], effects that would be expected to limit AAA growth [1, 8, 51]. Participants will be instructed to avoid ingesting aperients or coffee within 1 h of taking the study supplement because these may interfere with myo-inositol absorption [52]. Participants in the control group will receive four 500-mg capsules of inert cellulose powder, which is to be taken orally at 08:00 and 20:00 daily. It will be packaged in an identical fashion to the intervention drug by Pharmaceutical Packaging Professionals (Thebarton, Australia), an established current good manufacturing practice facility.

### Participant time line

**Fig. 2:**
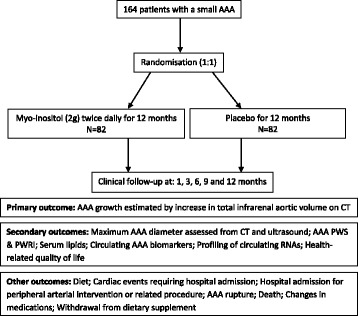
Design of the Inositol in the MAnaGemENt of abdominal aortic aneurysm (IMAGEN) trial. *AAA* Abdominal aortic aneurysm, *CT* Computed tomography, *PWRI* Peak wall rupture index, *PWS* Peak wall stress

The overall design of the IMAGEN trial is shown in Fig. 2. At vascular outpatient clinics, potential participants will be identified and pre-screened for suitability, and, if appropriate, informed consent will be obtained, after which they will be formally assessed against the eligibility criteria (Table 1). Individuals will undergo a medical examination, anthropometric assessment (height, weight, waist and hip circumference), resting blood pressure, heart rate and ankle-brachial index assessments, questionnaires (health-related quality of life and food frequency), a 24-h dietary recall survey, and collection of blood samples for measurement of full blood count (haemoglobin, red cell count, white cell count, platelets, neutrophils, lymphocytes, monocytes, eosinophils, basophils), urea and electrolytes (sodium, potassium, creatinine, estimated glomerular filtration rate, albumin, total bilirubin, urea, chloride, bicarbonate), liver function tests (alanine transaminase, aspartate aminotransferase, γ-glutamyl transpeptidase, lactate dehydrogenase), fasting lipids (cholesterol, triglyceride, HDL cholesterol and low-density lipoprotein [LDL] cholesterol), and fasting glucose. Serum, plasma and whole blood will also be collected for assessment of circulating proteins and genetic (DNA and RNA) analyses. Investigational blood samples will be collected into the following tubes: 2 x 5 ml SST, 2 x 4 ml ethylenediaminetetraacetic acid, 1 x 4 ml Sodium Citrate and 1 x 2.5 ml PAXgene (Qiagen, Valencia, CA, USA). Blood samples will be processed according to standard operating procedures and shipped to the study centre in Townsville. Baseline CT and ultrasound will be performed for detailed measurements of AAAs.

Eligible and consenting patients will be randomised to receive either 2 g of myo-inositol or identical placebo twice daily for 12 months. Participants will be contacted by telephone call at 1 month after commencing the trial medication and asked about adherence to the study drug regimen as well as adverse and clinical events. Phone consultations will be repeated at 3 and 9 months. Follow-up visits will occur at 6 and 12 months, when participants will receive ongoing questionnaires, clinical examinations, blood tests and imaging according to Fig. 1. The occurrence of adverse and clinical events and adherence to the study drug regimen will also be assessed. All participants, including those in whom medication is ceased, will be invited to follow-up visits for the duration of the study in the absence of AAA repair. Final follow-up will occur at 12 months after commencement of medication, at which point the participant will discontinue the prescribed medication and return any unused medication.

### Compliance

Previous studies suggest that compliance with dietary supplementation of 4 g of myo-inositol daily is excellent [19, 45]. To facilitate compliance, a number of techniques will be used, including providing participants with the telephone number of the local trial coordinator with instructions to contact in the event of possible supplement-related problems or consideration of discontinuation. In this event, arrangements will be made for the participant to be reviewed by the study physician to ascertain if discontinuation is required. The trial will also use a reminder system to facilitate compliance. This will consist of reminders from the local trial coordinator to each participant at 1, 3, 6 and 9 months after randomisation to check and encourage compliance. Participants will be provided with a compliance diary at the commencement of the trial. Participants will bring their diaries and remaining supplement capsules to review appointments at 6 and 12 months, and compliance will be checked by capsule and bottle counting. Where < 95% of capsules were taken since the last review, the study coordinator will attempt to work with the participant to identify the issues which have impacted compliance and provide suggestions on how to overcome these issues in future.

### Outcome assessment

Outcome assessment will be performed at baseline, 6 and 12 months. Participants will be imaged by ultrasound at baseline, 6 and 12 months and by aortic CT at baseline and 12 months. Blood will be collected at baseline, 6 and 12 months and will be used to assess biomarker outcomes and safety.

### Primary efficacy endpoint

The primary outcome measure will be AAA growth estimated by increase in total infrarenal aortic volume on the basis of CT. Previous studies indicate this is a reproducible and sensitive measure of AAA growth [53]. A standardised and established CT imaging protocol will be employed at all four centres, as previously described [53–55]. All CT images will be transferred to the central imaging centre in Townsville, where image quality will be confirmed. Total infrarenal aortic volume will be measured from the level directly below the renal arteries to the bifurcation using the Philips IntelliSpace Portal 7.0 (Philips Clinical Informatics, Amsterdam, The Netherlands), as previously described [53]. Central reading of the CT images will be performed by a single experienced observer who has previously been shown to have excellent imaging analysis reproducibility. The repeatability of central readings will be checked intermittently during the central reading period, which will span the duration of the trial.

### Secondary efficacy endpoints

Secondary outcome measures reflect the proposed mechanism of action of myo-inositol, are important and relevant clinical events, and are implicated in AAA growth.

#### Maximum AAA diameter assessed by CT

Maximum AAA diameter is the most validated marker of AAA rupture risk and currently the main determinant of when patients are selected for intervention [4–7, 56]. Maximum outer wall to outer wall AAA diameter will be assessed in anteroposterior (AP) and transverse orthogonal planes centrally from reconstructed aortic CT images obtained using a previously published reproducible protocol [53, 57–59]. The assessment will be performed by a single experienced observer who has previously been shown to have excellent imaging analysis reproducibility.

#### Maximum AAA diameter assessed by ultrasound

Participants will be assessed by ultrasound in the morning, having fasted from midnight. Maximum outer wall–to-outer wall diameter assessed in an AP orthogonal plane will be measured by experienced sonographers using a 3.75-MHz transducer and ultrasound machines present in the vascular laboratories of each centre (Toshiba Capasee, Toshiba Medical Systems, Duluth, GA, USA; Philips HDI 5000, Philips Medical Systems, Norcross, GA, USA; LOGIQ E9, GE Healthcare Life Sciences, Chicago, IL, USA), as previously described [44, 60]. The maximum AP inner wall-to-inner wall, leading edge wall-to-leading edge wall and outer wall-to-outer wall measurements on the largest AAA ultrasound image recorded at each time point will be centrally assessed by a single experienced observer who has previously been shown to have excellent imaging analysis reproducibility.

#### AAA PWS and PWRI

These parameters will be estimated by a single investigator using the A4research software (VASCOPS GmbH, Graz, Austria) with a reproducible and previously validated technique [2, 61, 62]. A 3D reconstruction of the AAA is produced from the CT images, and a fine element analysis mesh is automatically generated by the software. This is used to estimate the PWS and PWRI, providing a surrogate marker of rupture risk [61, 62].

#### Serum lipids

Total cholesterol, triglycerides, LDL cholesterol and HDL cholesterol concentrations will be assessed using automated assays (Hitachi 917; Roche Diagnostics GmbH, Mannheim, Germany) with serum obtained from patients who have fasted overnight. The inter-assay coefficient of variation (CV) for these assays has been shown to be between 2% and 5% in our previous studies [49, 50].

#### Circulating AAA biomarkers

The following biomarkers have been selected on the basis of their reported association with AAA and previous data suggesting they may be favourably modified by myo-inositol supplementation [16–19, 23–46, 63, 64]: serum resistin, plasma interferon γ, plasma CCL22, plasma D-dimer, plasma MMP-2, plasma MMP-9, plasma cathepsin S, serum OPN, plasma OPG and serum SOST. Concentrations will be measured using established commercial enzyme-linked immunosorbent assays (R&D Systems, Minneapolis, MN, USA) according to the manufacturer’s instructions and expressed as nanograms per millilitre. Intra-assay and inter-assay CV for all these assays are between 3% and 6% in our laboratory (concordance correlation coefficient ~ 0.999) [32, 35, 44, 47, 63–65].

#### Profiling of circulating RNAs

Blood will be collected into PAXgene tubes at baseline and at 6 and 12 months. RNA will be extracted from these samples and used to assess the expression profile of circulating cells. Established techniques will be used to assess expression profile, including microarrays, next-generation sequencing and RT-PCR as we have previously reported [66–68].

#### Health-related quality of life

Health-related quality of life will be assessed using the 36-item Short Form Health Survey (SF-36) completed at 0, 6 and 12 months and has previously been validated for use in patients with AAA [69]. Additional outcome measures include dietary analysis assessed by means of a food frequency questionnaire and 24-h recall interview at baseline and at 6 and 12 months, as previously described [70]. Finally, acute cardiovascular events, requirement for peripheral arterial intervention, AAA rupture, all-cause mortality, changes in background medication and withdrawal from the study dietary supplement (by indication) will be recorded. These data will be recorded through patient interview every 6 months, chart reviews and linked data, as previously described [71–73].

### Participant safety

Previous trials using dietary supplementation with 4 g of myo-inositol daily suggest it is associated with no ill effects. There have been no reports of excess gastrointestinal, cardiovascular, respiratory or cutaneous symptoms in those randomised to myo-inositol compared with placebo [16, 17, 19, 45]. Patient safety will be assessed at 6 and 12 months following randomisation by assessment of blood pressure and heart rate; laboratory parameters, including serum chemistry (liver function tests, glucose, creatinine, sodium, potassium, chloride, bicarbonate, calcium and phosphorous) and haematology (haemoglobin, differential white blood cell and platelet counts); and questions regarding adverse events (AEs). Participants will be contacted by telephone at 1, 3 and 9 months following randomisation to enquire about AEs. Furthermore, participants will be provided with full contact details of the site trial coordinators with instructions to contact them in the event of any AEs. All AEs will be reported via standardised forms to the coordinating centre and carefully monitored throughout the study. Serious adverse events (SAEs) will be defined according to current Australian GCP guidelines and will be reported according to the National Health and Medical Research Council (NHMRC) Safety Monitoring and Reporting in Clinical Trials Involving Therapeutic Goods guidelines (November 2016) [74–77]. All SAEs will be reviewed by the safety committee, which will consist of two independent vascular surgeons. The safety committee will concentrate on events which could be AAA-related, such as sudden death due to AAA rupture, or events that could be related to myo-inositol, such as gastrointestinal problems or electrolyte disturbances. Expected AAA rupture rates for small AAAs are ~ 1–2% per year [4–7]. Rupture rates > 5% per year will be considered cause for concern. In this instance, all other outcome data from the study will be analysed. AAA growth will be compared between groups using these data. The final decision to stop the study will take into account AAA ruptures, AAA growth data and any other serious complications. On the basis of findings, the safety committee will report their decision to the steering committee.

### Interim analysis

An interim analysis assessing trial efficacy will be conducted once approximately one-third of participants have completed 12 months of myo-inositol treatment. In this analysis, we will assess changes in AAA volume and diameter over time to determine whether (a) a suggested difference in AAA growth between participant groups is observed and (b) whether a priori calculated sample sizes will provide adequate power to detect any observed differences in AAA growth. These findings will be conducted by an independent statistician using dummy-coded data to separate patients into drug or placebo groups (e.g., X and Y), thereby maintaining trial blinding and minimising potential bias. In the instance of a clear difference in AAA growth rate between groups, the statistician will liaise with the dispensing pharmacist to determine if participants receiving inositol exhibit faster AAA growth than control participants. Findings from this interim analysis will be reported to members of the steering committee, who will consider concluding the trial if either (a) myo-inositol administration appears to be associated with accelerated AAA growth (ethical assessment) or (b) revised sample size calculations based on observed AAA growth for the cohort suggest that group sizes need to be increased beyond what can reasonably be achieved (futility assessment).

### Sample size and power calculations

It is hypothesized that dietary supplementation with 4 g of myo-inositol daily for 12 months will reduce AAA growth by 40%, as evidenced by limiting increase in AAA volume from 8.1 ± 6.9 cm^3^ to 4.9 ± 6.9 cm^3^. Estimated outcomes for the control group are based on CT volume measured in a pilot cohort [53]. An effect size of 40% is based on the consideration of outcomes likely to be required for clinical efficacy. For example, if myo-inositol reduced annual increase in AAA size by 40%, the estimated time for an AAA initially measuring 40 mm to reach the commonly employed intervention level of 50 mm would be increased from 5 to ~ 7 years. Given that the annual mortality rate due to non-AAA-related causes is ~ 5% in patients with small AAAs, this reduction in growth could be expected to result in ~ 10% less patients requiring interventions over 5 years. This has significant potential social and economic benefit, with a possible cost saving of ~ $1 million per year [5, 6, 12]. On the basis of these estimated outcomes, 74 participants per group are required (80% power, α = 0.05) to detect the hypothesized reductions in AAA volume. Using data from a pilot cohort, it is estimated that this sample size will have a power of ~ 85% to detect a 40% reduction in orthogonal AAA diameter increase, assuming that the SD for the intervention group reduces in line with the mean. Allowing for a dropout rate of 10%, a total of 164 patients will be required. Sample sizes were calculated using G*Power 3.1 software.

### Data analysis

Analysis of primary and secondary endpoints will be based on intention to treat at the time of randomisation. All participants who meet the eligibility criteria, provide written informed consent and are enrolled in the study will be included in the primary analysis, regardless of adherence to supplement allocation. The efficiency of randomisation will be assessed by comparing the distribution of recognized determinants of AAA growth, such as initial AAA diameter, and background medication between the intervention and control groups.

The primary efficacy parameter is the between-group difference in mean AAA growth over 12 months as estimated by infrarenal aortic volume measured by CT. If there were to be no dropouts, the observed mean AAA volume increase over 12 months would be compared using a two-sample *t* test. However, it is expected that 10% of participants will drop out. AAA growth will therefore be analysed using a linear mixed model, specifying zero difference between treatment groups at baseline. The model will have two repeated dependent measurements. The fixed part of the model will contain the following covariates: time for CT measurement as a categorical variable, treatment, and interaction between treatment and CT time. The covariance part of the model will constitute a linear covariance matrix containing the following parameters: variance of CT measurements and covariance parameters. Without reference to the fixed part of the model, the goodness of fit of the assumed covariance model will be investigated. If necessary, the model will be extended with additional terms. The analysis will be performed using the PROC MIXED procedure in SAS software (SAS Institute, Cary, NC, USA), which is capable of fitting arbitrary linear covariance matrices. The main null hypothesis that will be tested is based on the treatment effect as estimated by this model at 12 months on CT measurements.

Secondary outcomes, including AAA diameter (CT and ultrasound), AAA PWS and PWRI, serum lipids, AAA biomarkers, RNA expression profile and SF-36 scores will be compared between the intervention and control groups using a linear mixed effect model similar to that described for the primary outcome. A number of additional analyses will also be performed, depending on the results of the primary analyses described above. These may include adjusted analyses using linear and logistic regression, such as where any important difference between the intervention and control groups are identified and Kaplan-Meier analyses to compare cardiovascular events and AAA repair rates.

### Trial and data organisation

This project has been developed through collaboration between experts in the biology and clinical management of AAA, aortic imaging and clinical trials. The trial steering committee comprises senior vascular investigators from the centres involved and experts in drug trials and data analysis. This will be the main policy- and decision-making committee for the study and will meet by teleconference on a quarterly basis. The clinical trial randomisation centre will be located at the NHMRC Centre for Cardiovascular Research & Education in Therapeutics at Monash University. A study coordinator located at James Cook University will coordinate activities across all the participating sites, assisting with local ethics reports, and communicating with the local sites, including updates and maintenance of the trial website, central computerised randomisation, data collection, entry and processing. Trial coordination will be facilitated by a clinical trial network established by the National Health and Medical Research Council-funded Centre of Research Excellence for Peripheral Arterial Disease. To determine whether important differences in the incidence of significant AEs exist between the treatment arms, an independent safety committee has been formed. The core imaging/biomarker laboratory located in Townsville will receive DICOM images of the CT scans and frozen serum/plasma to assess the CT and biomarker outcomes. Baseline and follow-up data will be collected on CRFs. All data will be stored in a central database and examined for data quality. A publications committee will oversee the preparation of reports arising from the study.

## Discussion

One of the main risk factors for AAA is age, with the problem being much more common in the > 60-year-old age group [1, 8]. With the continued expansion in the elderly population, the number of AAA operations can be expected to continue to increase [78]. Alternative forms of treatment for AAA are urgently needed, ideally those which are non-invasive, suitable for the older age group and cost-effective. The IMAGEN trial will be the first randomised trial, to our knowledge, to assess the value of myo-inositol in limiting AAA growth. To date, only two trials of medical treatment of AAA with sufficient sample size have been published, and both had negative findings [9, 79]. If the present hypotheses are confirmed, it is estimated that myo-inositol could lead to a reduction in AAA surgery of ~ 1–2% per year at the expense of a low-cost dietary supplement. The results of this trial could markedly change the clinical management of small AAAs, which currently consists of ultrasound surveillance. This study will also provide important information on (a) biomarkers which predict responsiveness to myo-inositol, (b) the effect of myo-inositol on cardiac events and (c) biomarkers for AAA growth. The IMAGEN trial may identify circulating markers which can be used to guide management of small AAA and selection for therapy by drugs or surgical interventions.

### Trial status

Participants are currently being recruited.

## Additional file

Additional file 1: SPIRIT 2013 checklist: recommended items to address in a clinical trial protocol and related documents. (DOC 122 kb)

## Abbreviations

AAA: Abdominal aortic aneurysm
ABI: Ankle-brachial index
AE: Adverse event
AP: Anteroposterior
BP: Blood pressure
CCL22: C-C motif chemokine 22
CRF: Case report form
CT: Computed tomography
CV: Coefficient of variation
GCP: Good clinical practice
HDL: High-density lipoprotein
IMAGEN: Inositol in the MAnaGemENt of abdominal aortic aneurysm
LDL: Low-density lipoprotein
MMP: Matrix metalloproteinase
mTOR: Mechanistic target of rapamycin
NF-κB: Nuclear factor kappa-light-chain-enhancer of activated B cells
NHMRC: National Health and Medical Research Council
OPG: Osteoprotegerin
OPN: Osteopontin
PWRI: Peak wall rupture index
PWS: Peak wall stress
SAE: Serious adverse event
SF-36: 36-item Short Form Health Survey
SOST: Sclerostin
SPIRIT: Standard Protocol Items: Recommendations for Interventional Trials
